# Revisiting foraging approaches in neuroscience

**DOI:** 10.3758/s13415-018-00682-z

**Published:** 2019-01-03

**Authors:** Sam Hall-McMaster, Fabrice Luyckx

**Affiliations:** 1 Department of Experimental Psychology, New Radcliffe House, Radcliffe Observatory, University of Oxford, Oxford, OX2 6HG UK; 2 Oxford Centre for Human Brain Activity, Department of Psychiatry, Warneford Hospital, University of Oxford, Oxford, OX3 7JX UK

**Keywords:** Foraging theory, Sequential decision-making, Reward-maximization

## Abstract

Many complex real-world decisions, such as deciding which house to buy or whether to switch jobs, involve trying to maximize reward across a sequence of choices. Optimal Foraging Theory is well suited to study these kinds of choices because it provides formal models for reward-maximization in sequential situations. In this article, we review recent insights from foraging neuroscience, behavioral ecology, and computational modelling. We find that a commonly used approach in foraging neuroscience, in which choice items are encountered at random, does not reflect the way animals direct their foraging efforts in certain real-world settings, nor does it reflect efficient reward-maximizing behavior. Based on this, we propose that task designs allowing subjects to encounter choice items strategically will further improve the ecological validity of foraging approaches used in neuroscience, as well as give rise to new behavioral and neural predictions that deepen our understanding of sequential, value-based choice.

## Introduction

For many decades, neural processes governing reward maximization have been a central interest in decision neuroscience. One framework that has contributed to our understanding of these processes is reinforcement learning (RL), in which an animal learns to maximize long-run future reward by trial-and-error (Sutton & Barto, 1998) or model-based strategies (Lee, Shimojo, & O’Doherty, 2014). As one example of this contribution, studies in RL have led to the proposal that midbrain dopamine neurons convey a prediction error signal to dorsal anterior cingulate cortex (dACC), allowing it to influence motor control selection when errors are made, or reward outcomes are lower than expected (Holroyd & Coles, 2002; Holroyd et al., 2004; Silvetti, Alexander, Verguts, & Brown, 2014).

In recent years, RL has been complimented by a new approach, based on Optimal Foraging Theory (Stephens & Krebs, 1986). The foraging approach has two main characteristics. The first is that it focuses on optimization problems, in which subjects must maximize a reward quantity in relation to another variable, such as time or effort expended. The second is that foraging tasks consist of a foreground option, which is currently being pursued, and background options the subject is not currently engaged with (Hayden, 2018; Hayden & Walton, 2014; Pearson, Watson, & Platt, 2014; Stephens, 2008). The foraging approach is thus distinct from many RL and economic choice tasks, in which choice items are presented simultaneously (Mobbs, Trimmer, Blumstein, & Dayan, 2018; Pearson et al., 2014). In addition, most foraging studies in neuroscience do not allow decision-makers to direct their encounters with choice items, a feature that is distinct from many sequential RL tasks, in which choice items or bandits can be revisited through model-based planning (Daw, Gershman, Seymour, Dayan, & Dolan, 2011; Kool, Gershman, & Cushman, 2017; Mobbs et al., 2018).

Two long-standing theoretical problems in animal behavior research have guided the recent interest in foraging within neuroscience (e.g., Kolling, Behrens, Mars, & Rushworth, 2012; Pearson et al., 2014). The first deals with *stay-switch* (or patch-leaving) dilemmas, in which an individual experiences an option with diminishing returns and must decide when it is best to leave that option. The other deals with *accept-reject* (or diet-selection) dilemmas, in which an individual chooses whether to engage with an option or ignore it in search of a better one (Stephens & Krebs, 1986; Stephens, 2008).

Recent applications of foraging approaches in neuroscience have drawn on foundational models in Foraging Theory (Charnov, 1976; Stephens & Krebs, 1986), generating new avenues and insights in the study of sequential, value-based choice (e.g., Hayden et al., 2011; Kolling et al., 2012). Nevertheless, growing evidence from behavioral ecology demonstrates that these approaches do not capture situations in which animals can use their knowledge of the environment to encounter choice items strategically (Fagan et al., 2013; Janmaat, Ban, & Boesch, 2013; Menzel, 1999; Merkle, Potts, & Fortin, 2017; Merkle, Fortin, & Morales, 2014; Sayers & Menzel, 2012). The purpose of this article is to review current approaches used in foraging neuroscience, to present evidence that these approaches are violated by animals in some settings, and to provide practical recommendations that offer new scope for understanding sequential, value-based choice.

### Insights from foraging neuroscience

Neural investigations using stay-switch and accept-reject tasks have primarily revealed important contributions of dACC to decision-making. Research using the stay-switch framework has shown that firing rates of neurons in monkey dACC positively correlate with decisions to leave a patch (option disengagement). These firing rates scale with increasing travel time to the next patch, suggesting that dACC neurons may set a threshold for switching and that this threshold is sensitive to the time cost of switch decisions (Hayden et al., 2011). Research using the accept-reject framework has shown that neurons in monkey dACC encode the time cost associated with choosing an option (option engagement). The activity patterns in a subset of dACC neurons also correlate with the value of rejected offers, suggesting that dACC keeps track of post-decision variables (Blanchard & Hayden, 2014).

In addition to insights from non-human primates, accept-reject studies in humans have played an important role in functional comparisons between dACC and the ventromedial prefrontal cortex (vmPFC) (Rushworth, Kolling, Sallet, & Mars, 2012). In particular, studies comparing accept-reject choices with economic ones have shown that BOLD activity in the dACC is positively correlated with the value of searching for alternative options, whereas vmPFC activity is positively correlated with the chosen option’s reward magnitude during binary economic choices (Kolling et al., 2012). These results seem to suggest that dACC plays a more prominent role in decisions to engage with options or search for alternatives and that the vmPFC plays a more prominent role in classic binary economic decisions. However, more recent work has provided evidence for another interpretation of dACC function in foraging scenarios, wherein dACC BOLD activity reflects choice difficulty (Ebitz & Hayden, 2016; Shenhav, Straccia, Cohen, & Botvinick, 2014). Additionally, BOLD signals in dACC and vmPFC have been shown to correlate with the relative value of searching for alternatives over engaging with current options, suggesting that both regions may represent similar information in foraging scenarios (Shenhav, Straccia, Botvinick, & Cohen, 2016b).

Alongside ongoing debate about frontal contributions to foraging, two major trends are emerging in foraging neuroscience. One is that we are beginning to expand our knowledge of foraging circuitry beyond the dACC and vmPFC (Barack, Chang, & Platt, 2017; Kane et al., 2017; Lottem et al., 2018). For example, it has recently been shown that neurons in posterior cingulate cortex (PCC) ramp up activity several seconds before monkeys make switch decisions, suggesting PCC signals reflect either an increasing motivation to disengage from current options, the value of alternative options or choice difficulty (Barack et al., 2017). In addition, recent evidence demonstrates that pharmacogenetically increasing neuronal activity in the locus coeruleus, a major site of norepinephrine neurons, causes rats to make switch decisions significantly earlier than control animals (Kane et al., 2017). By contrast, optogenetic activation of neurons in the dorsal raphae nucleus, a major site of serotonin neurons, causes mice to persist with active exploitation decisions for longer and abandon patches later than controls (Lottem et al., 2018). Taken together, these results suggest that several regions beyond dACC, including subcortical structures with distinct neurotransmitter classes (catecholamines and monoamines), make important contributions to the neural computations performed during stay-switch dilemmas and the choices that result.

The second major trend is the increasing use of RL techniques in foraging research, to model behavior in environments where on-going learning is needed to optimize decisions (Constantino & Daw, 2015; Frankenhuis, Panchanathan, & Barto, 2018; Kolling & Akam, 2017; Wittmann et al., 2016). One classic model for optimal stay-switch behavior in foraging is called the marginal value-theorem (MVT), which states that, for environments with patches that have monotonically depleting reward rates, it is optimal to leave the current patch when its reward rate drops to the average reward rate of the environment (Charnov, 1976). Constantino and Daw (2015) showed that MVT better predicts trial-by-trial choices in a stay-switch task than a temporal difference RL model. However, when patches are not monotonically depleting or people need to anticipate future values within a patch, models using RL parameters can provide more optimal solutions (Kolling & Akam, 2017). For instance, an RL model that generates value estimates for stay-switch choices by integrating reward information over multiple time scales successfully predicted whether people would switch away from current options in a dynamic patch environment, and components of this model were reflected in BOLD activity of human dACC (Wittmann et al., 2016).

### The case for directed encounters

In many of the foraging studies reviewed above, subjects have limited (and often no) control over the successive items they encounter (Constantino & Daw, 2015; Hayden et al., 2011; Kolling et al., 2012; Shenhav et al., 2016b; Shenhav et al., 2014). In other words, new prey and patch items are encountered randomly or pseudorandomly. The use of this approach in neuroscience has undoubtedly deepened our understanding about foraging behavior and sequential decision-making more broadly (e.g., Hayden et al., 2011; Kolling et al., 2012). Nevertheless, mounting evidence from studies in behavioral ecology indicate that designs with purely random encounters do not reflect situations in which animals can direct their foraging efforts by choosing to revisit valuable patches or prey items. To illustrate, consider a fruit tree that contains a small portion of ripe fruit and a large portion of unripe fruit. The ripe fruit can be harvested immediately but the animal may also benefit from revisiting the tree to harvest the remaining fruit once it ripens. Similarly, it may be beneficial to remember the locations of trees bearing a large amount of fruit in the summer months, so they can be exploited the following season. This kind of foraging strategy, in which high value items are revisited and low value items are avoided, could greatly reduce search time and thereby improve biological fitness (Merkle et al., 2017, 2014; Riotte-Lambert, Benhamou, & Chamaillé-Jammes, 2015; Sayers & Menzel, 2012).

A substantial body of empirical work corroborates the notion that, in several situations, animals do not encounter choice items at random but use their knowledge of the environment to encounter items strategically (Fagan et al., 2013). Bison move to meadows that have previously been profitable, especially after encountering a poor quality patch (Merkle et al., 2014). When modelling bison patch selection, a model using random patch encounters is less efficient than a model incorporating memory processes, and cannot fully capture bison choice behavior (Merkle et al., 2017; see also Riotte-Lambert et al., 2015). In non-human primates, chimpanzees have been observed to monitor candidate fruit trees during travel but also engage in goal-directed travel to specific trees, based on the size and location of trees they have learned about from previous visits (Janmaat et al., 2013). Alongside real-world studies, more controlled laboratory experiments indicate that when animals are allowed to direct their search strategy, they are unequivocally non-random. Chimpanzees direct their search by integrating information about food quantity, handling time, and distance from previous encounters to maximize the rate of energy intake (Sayers & Menzel, 2012), and rhesus monkeys rarely revisit patches they know to be empty from previous experience, but do so when memory is impaired by lesioning the sulcus principalis (Passingham, 1985).

Situations allowing animals to direct their encounters with choice items introduces a new decision dilemma. In stay-switch situations, for example, animals must not only decide *when* to disengage from the current option, but also *where* to move next. In natural environments, this selection dilemma may be especially relevant to central-place foragers, such as bees or owls, who undertake foraging trips from a central hive or nest. Evidence suggests that patch-selection choices of central-place foragers are not random (Rosenberg & McKelvey, 1999) and that directed selection-based knowledge of the environment can increase reward intake when patches are spatially clustered (Barraquand, Inchausti, & Bretagnolle, 2009). This selection dilemma may be less important to inter-patch (or ‘nomadic’) foragers, who are often assumed to encounter patches or prey at random (Barraquand et al., 2009).

### Practical suggestions

Based on the evidence outlined above, one way to extend foraging approaches in neuroscience is to allow non-random encounters in future designs. For illustrative purposes, we can imagine a simple grid-world task as one possible assay. The grid contains a number of randomly distributed ‘patch’ tiles, from which rewards can be harvested and a number of intervening tiles, which represent the travel time between patches. Following a leave decision, participants select the next patch tile they wish to visit, which could include both previously visited patches and unexplored ones. To provide context to the predictions that follow, we assume patches have different maximum reward values but follow the same depletion function when being exploited. We also assume the maximum reward capacity of each patch is fixed and that subjects’ patch selections are deterministic. Our final assumption is that the replenish rate of patches exceeds the travel time to reach them. Under this final assumption, MVT converges to the optimal solution (Possingham & Houston, 1990), making it an adequate predictive framework for our purposes.

With these changes in mind, the empirical work reviewed above implies several behavioral and neural predictions. Behaviorally, one prediction is that patch selection will become less random over time. From an RL perspective, this can be viewed as a declining policy parameter that reduces the amount of exploration relative to exploitation (Sutton & Barto, 1998). As subjects learn about the value and distribution of different patches, they can encounter items strategically by revisiting high-value items, which have high reward-travel time ratios. Once patch values have been learnt, designs allowing directed encounters should produce lower switching thresholds than random encounter designs. From the perspective of MVT, choosing only options with high reward-travel time ratios will increase the average experienced reward rate, allowing this *global* switching threshold to be met more quickly by the current option. From another perspective, subjects may set their leaving threshold to the next best alternative (i.e., a *local* threshold set to the alternative with the highest reward-travel time ratio) in a directed encounter design. Because low value options can be avoided, this local threshold would also be met more quickly by the current option. Whether subjects use a global or local leaving threshold in directed designs is an empirical question. For our purposes, what is most important is that both perspectives predict lower leaving thresholds, compared with a random encounter design in which MVT provides the optimal solution.

From a neural perspective, if the firing rate of dACC neurons reflects a threshold for switching (Hayden et al., 2011), dACC thresholds for stay-switch decisions should be lower in task designs that allow directed encounters compared with random encounter designs. Again, this is because repeated exposure to low-value alternatives can be avoided in directed encounter designs, resulting in a lower global or local threshold for switching away from current options. This prediction has parallels with Hayden et al. (2011), who manipulated reward rates by changing the travel time between patches. This work showed that dACC thresholds for switching were lower for decreasing travel times (i.e., higher reward rates), consistent with the proposal that higher reward rates afforded by directed encounter designs should produce lower leaving thresholds.

## Discussion

These suggestions represent a first step towards the development of new foraging approaches in neuroscience, which allow directed encounters with choice items. More complex designs could consider different relationships between replenish rate and travel time. The importance of these factors has been highlighted by Possingham and Houston (1990), who show that when patches replenish slower than the time it takes to revisit them, MVT fails to maximize reward. This has also been demonstrated in stochastic environments, where repeated sampling is required to determine patch quality, or environments where reward rates initially increase as the richest part of the patch is located (McNamara, 1982; Oaten, 1977). In these latter situations, Kolling and Akam (2017) propose that comparing the expected future reward rate of a patch against the global average will lead to more optimal patch-leaving choices than traditional MVT (Charnov, 1976). Thus, RL frameworks may be increasingly important for understanding choice behavior in more complex foraging designs, such as those that allow patch revisiting.

In addition to these considerations, dACC has many competing theories about its role in decision-making. As a starting point, we have framed our neural predictions under the view that dACC may encode a threshold for switching away from current options (Hayden et al., 2011). A related view proposes that dACC modulates behavioral flexibility by tracking the value of alternative choice options (search value) (Kolling, Behrens, Wittmann, & Rushworth, 2016a; Kolling et al., 2016b). These accounts may not be mutually exclusive (Blanchard & Hayden, 2014); however, it remains an open question whether dACC activity will be lower at the point switches are made, reflecting a lower switching threshold (Hayden, 2011), or whether it will be higher due to higher search values (Kolling et al. 2012; Kolling et al., 2016a, b) in directed encounter designs. In contrast to accounts focusing on dACC’s role in behavioral persistence and flexibility, Expected Value of Control Theory contends that dACC signals the need for cognitive control and the value of exerting control, given its computational cost. By integrating this information, the theory ascribes dACC a key role in determining where cognitive control should be directed and how much it should be deployed (Ebitz & Hayden, 2016; Shenhav, Cohen, & Botvinick, 2016a; Shenhav et al., 2014). Single-cell recordings and anatomical studies provide evidence for another view, wherein dACC tracks contextual variables, linking task contexts with appropriate action plans (Heilbronner & Hayden, 2016). Foraging studies have played a prominent role in contemporary debate about dACC (Ebitz & Hayden, 2016) and the development of new foraging approaches, such as those involving dynamic replenish rates or allowing patch revisiting, may be useful to further distinguish competing theories about its function.

In this respect, one limitation of the stay-switch designs we have covered is that there may be an insufficient range of foreground and background values to fully decouple flexibility (Kolling et al., 2016b) from choice difficulty accounts (Shenhav et al., 2016a; Shenhav et al., 2014). Subjects are unlikely to stay in patches until the current value is dramatically lower than the average experienced reward rate, whereas it is much easier to present a low value foreground offer against a high value set of alternatives in accept-reject studies. The use of directed encounters in accept-reject designs may thus be useful in considering further tests of dACC theories. In such designs, participants could have the choice about whether to engage with a two-option foreground gamble, with a set of alternatives visible in the background (Kolling et al., 2012; Shenhav et al., 2016b; Shenhav et al., 2014). Rejecting the foreground offer, the subject would be able to select an option from a set of background alternatives as one of the next foreground options, in contrast to previous studies where the new foreground options were randomly drawn from the set of alternatives. In a similar manner to stay-switch designs, one prediction is that rejection thresholds will no longer be set based on the average value of the alternatives (a global threshold) but on the most valuable alternative among the set (a local threshold). Note that in this case the global threshold refers to the average value of presented alternatives, whereas in stay-switch designs it refers to the average experienced reward rate. If dACC activity correlates with choice difficulty (Shenhav et al., 2016a, b; Shenhav et al., 2014), we should observe a difference in activity for the same set of alternatives. Specifically, if the value of the alternatives being considered are different under random and directed encounter conditions (e.g., background average vs. best alternative), this would make the decision to reject more or less difficult, and this difference should be reflected in dACC activity under the choice difficulty account. Therefore, while many of our practical suggestions have been framed under the broad view that dACC contributes to behavioral flexibility (Hayden et al., 2011; Kolling et al., 2012; Kolling et al., 2016b), the example above shows that directed encounters can be applied to other theories of dACC. Moreover, while our neural predictions have focused on dACC, the development of new foraging approaches, such as those allowing options to be revisited, may hold value in supporting the recent trend of investigating other cortical and subcortical contributions to foraging decisions (Barack, Chang, & Platt, 2017; Kane et al., 2017; Lottem et al., 2018).

## Conclusion

Neuroscience has made great strides in using foraging as a tool to understand the neural computations that underlie sequential, value-based choice. In reviewing these insights, alongside those from behavioral ecology and computational modelling, we have presented evidence demonstrating that, while useful, current task designs involving random encounters with choice items do not reflect situations in which animals can make use of their knowledge in the environment to encounter items strategically (Fagan et al., 2013; Menzel, 1999; Merkle et al., 2017, 2014; Passingham, 1985; Sayers & Menzel, 2012). Based on this evidence, we propose that task designs allowing subjects to revisit choice items will further improve the ecological validity of foraging approaches used in neuroscience. In addition, we have derived several behavioral and neural predictions centered on the idea that the value of switching away from current options can be higher in directed encounter designs. This is because individuals can base their decisions to switch on a subset of valuable alternatives, modulating global or local thresholds for choice disengagement. Finally, there is a clear trend towards integrating RL and foraging approaches to understand sequential choice processes in complex settings (Constantino & Daw, 2015; Kolling & Akam, 2017; Wittmann et al., 2016). The recommendations and predictions outlined in this review may be useful starting points for the development of increasingly naturalistic foraging designs, in which elements like patch revisiting, replenish rates and patch distributions are manipulated to further unravel contributions of learning and memory to sequential, value-based choice.

## References

[CR1] Barack DL, Chang SWC, Platt ML (2017). Posterior cingulate neurons dynamically signal decisions to disengage during foraging. Neuron.

[CR2] Barraquand F, Inchausti P, Bretagnolle V (2009). Cognitive abilities of a central place forager interact with prey spatial aggregation in their effect on intake rate. Animal Behaviour.

[CR3] Blanchard TC, Hayden BY (2014). Neurons in dorsal anterior cingulate cortex signal postdecisional variables in a foraging task. Journal of Neuroscience.

[CR4] Charnov EL (1976). Optimal foraging, the Marginal Value Theorem. Theoretical Population Biology.

[CR5] Constantino SM, Daw ND (2015). Learning the opportunity cost of time in a patch-foraging task. Cognitive, Affective & Behavioral Neuroscience.

[CR6] Daw ND, Gershman SJ, Seymour B, Dayan P, Dolan RJ (2011). Model-based influences on humans’ choices and striatal prediction errors. Neuron.

[CR7] Ebitz RB, Hayden BY (2016). Dorsal anterior cingulate: A Rorschach test for cognitive neuroscience. Nature Neuroscience.

[CR8] Fagan WF, Lewis MA, Auger-Méthé M, Avgar T, Benhamou S, Breed G (2013). Spatial memory and animal movement. Ecology Letters.

[CR9] Frankenhuis, W. E., Panchanathan, K., & Barto, A. G. (2018). Enriching behavioral ecology with reinforcement learning methods. *Behavioural Processes*. 10.1016/j.beproc.2018.01.008 29412143

[CR10] Hayden BY (2018). Economic choice: the foraging perspective. Current Opinion in Behavioral Sciences.

[CR11] Hayden BY, Pearson JM, Platt ML (2011). Neuronal basis of sequential foraging decisions in a patchy environment. Nature Neuroscience.

[CR12] Hayden BY, Walton ME (2014). Neuroscience of foraging. Frontiers in Neuroscience.

[CR13] Heilbronner SR, Hayden BY (2016). Dorsal anterior cingulate cortex: A bottom-up view. Annual Review of Neuroscience.

[CR14] Holroyd CB, Coles MGH (2002). The neural basis of human error processing: Reinforcement learning, dopamine, and the error-related negativity. Psychological Review.

[CR15] Holroyd CB, Nieuwenhuis S, Yeung N, Nystrom L, Mars RB, Coles HMG, Cohen JD (2004). Dorsal anterior cingulate cortex shows fMRI response to internal and external error signals. Nature Neuroscience.

[CR16] Janmaat KRL, Ban SD, Boesch C (2013). Chimpanzees use long-term spatial memory to monitor large fruit trees and remember feeding experiences across seasons. Animal Behaviour.

[CR17] Kane GA, Vazey EM, Wilson RC, Shenhav A, Daw ND, Aston-Jones G, Cohen JD (2017). Increased locus coeruleus tonic activity causes disengagement from a patch-foraging task. Cognitive, Affective and Behavioral Neuroscience.

[CR18] Kolling N, Akam T (2017). (Reinforcement?) Learning to forage optimally. Current Opinion in Neurobiology.

[CR19] Kolling N, Behrens TEJ, Mars RB, Rushworth MFS (2012). Neural mechanisms of foraging. Science.

[CR20] Kolling N, Behrens TEJ, Wittmann MK, Rushworth MFS (2016). Multiple signals in anterior cingulate cortex. Current Opinion in Neurobiology.

[CR21] Kolling N, Wittmann MK, Behrens TEJ, Boorman ED, Mars RB, Rushworth MFS (2016). Value, search, persistence and model updating in anterior cingulate cortex. Nature Neuroscience.

[CR22] Kool W, Gershman SJ, Cushman FA (2017). Cost-benefit arbitration between multiple reinforcement learning systems. Psychological Science.

[CR23] Lee SW, Shimojo S, O’Doherty JP (2014). Neural computations underlying arbitration between model-based and model-free learning. Neuron.

[CR24] Lottem E, Banerjee D, Vertechi P, Sarra D, Lohuis MO, Mainen ZF (2018). Activation of serotonin neurons promotes active persistence in a probabilistic foraging task. Nature Communications.

[CR25] McNamara J (1982). Optimal patch use in a stochastic environment. Theoretical Population Biology.

[CR26] Menzel R (1999). Memory dynamics in the honeybee. Journal of Comparative Physiology - A Sensory, Neural, and Behavioral Physiology.

[CR27] Merkle JA, Fortin D, Morales JM (2014). A memory-based foraging tactic reveals an adaptive mechanism for restricted space use. Ecology Letters.

[CR28] Merkle JA, Potts JR, Fortin D (2017). Energy benefits and emergent space use patterns of an empirically parameterized model of memory-based patch selection. Oikos.

[CR29] Mobbs D, Trimmer PC, Blumstein DT, Dayan P (2018). Foraging for foundations in decision neuroscience: Insights from ethology. Nature Reviews Neuroscience.

[CR30] Oaten A (1977). Optimal foraging in patches: A case for stochasticity. Theoretical Population Biology.

[CR31] Passingham RE (1985). Memory of monkeys (Macaco mulatta) with lesions in prefrontal cortex. Behavioral Neuroscience.

[CR32] Pearson JM, Watson KK, Platt ML (2014). Decision making: The neuroethological turn. Neuron.

[CR33] Possingham HP, Houston AI (1990). Optimal patch use by a territorial forager. Journal of Theoretical Biology.

[CR34] Riotte-Lambert L, Benhamou S, Chamaillé-Jammes S (2015). How memory-based movement leads to nonterritorial spatial segregation. The American Naturalist.

[CR35] Rosenberg DK, McKelvey KS (1999). Habitat selection for central-place foraging animals. The Journal of Wildlife Management.

[CR36] Rushworth MFS, Kolling N, Sallet J, Mars RB (2012). Valuation and decision-making in frontal cortex: One or many serial or parallel systems?. Current Opinion in Neurobiology.

[CR37] Sayers K, Menzel CR (2012). Memory and foraging theory: Chimpanzee utilization of optimality heuristics in the rank-order recovery of hidden foods. Animal Behaviour.

[CR38] Shenhav A, Cohen JD, Botvinick MM (2016). Dorsal anterior cingulate cortex and the value of control. Nature Neuroscience.

[CR39] Shenhav A, Straccia MA, Botvinick MM, Cohen JD (2016). Dorsal anterior cingulate and ventromedial prefrontal cortex have inverse roles in both foraging and economic choice. Cognitive, Affective & Behavioral Neuroscience.

[CR40] Shenhav A, Straccia MA, Cohen JD, Botvinick MM (2014). Anterior cingulate engagement in a foraging context reflects choice difficulty, not foraging value. Nature Neuroscience.

[CR41] Silvetti M, Alexander W, Verguts T, Brown JW (2014). From conflict management to reward-based decision-making: Actors and critics in primate medial frontal cortex. Neuroscience and Biobehavioral Reviews.

[CR42] Stephens DW (2008). Decision ecology: Foraging and the ecology of animal decision-making. Cognitive, Affective & Behavioral Neuroscience.

[CR43] Stephens DW, Krebs JR (1986). Foraging theory.

[CR44] Sutton RS, Barto AG (1998). Reinforcement learning : An introduction.

[CR45] Wittmann MK, Kolling N, Akaishi R, Chau BKH, Brown JW, Nelissen N, Rushworth MFS (2016). Predictive decision-making driven by multiple time-linked reward representations in the anterior cingulate cortex. Nature Communications.

